# Identifying COVID-19 Outbreaks From Contact-Tracing Interview Forms for Public Health Departments: Development of a Natural Language Processing Pipeline

**DOI:** 10.2196/36119

**Published:** 2022-03-08

**Authors:** John Caskey, Iain L McConnell, Madeline Oguss, Dmitriy Dligach, Rachel Kulikoff, Brittany Grogan, Crystal Gibson, Elizabeth Wimmer, Traci E DeSalvo, Edwin E Nyakoe-Nyasani, Matthew M Churpek, Majid Afshar

**Affiliations:** 1 University of Wisconsin–Madison Madison, WI United States; 2 Loyola University Chicago Chicago, IL United States; 3 Public Health Madison & Dane County Madison, WI United States; 4 State of Wisconsin Department of Health Services Madison, WI United States

**Keywords:** natural language processing, public health informatics, named entity recognition, contact tracing, COVID-19, outbreaks, neural language model, disease surveillance, digital health, electronic surveillance, public health, digital surveillance tool

## Abstract

**Background:**

In Wisconsin, COVID-19 case interview forms contain free-text fields that need to be mined to identify potential outbreaks for targeted policy making. We developed an automated pipeline to ingest the free text into a pretrained neural language model to identify businesses and facilities as outbreaks.

**Objective:**

We aimed to examine the precision and recall of our natural language processing pipeline against existing outbreaks and potentially new clusters.

**Methods:**

Data on cases of COVID-19 were extracted from the Wisconsin Electronic Disease Surveillance System (WEDSS) for Dane County between July 1, 2020, and June 30, 2021. Features from the case interview forms were fed into a Bidirectional Encoder Representations from Transformers (BERT) model that was fine-tuned for named entity recognition (NER). We also developed a novel location-mapping tool to provide addresses for relevant NER. Precision and recall were measured against manually verified outbreaks and valid addresses in WEDSS.

**Results:**

There were 46,798 cases of COVID-19, with 4,183,273 total BERT tokens and 15,051 unique tokens. The recall and precision of the NER tool were 0.67 (95% CI 0.66-0.68) and 0.55 (95% CI 0.54-0.57), respectively. For the location-mapping tool, the recall and precision were 0.93 (95% CI 0.92-0.95) and 0.93 (95% CI 0.92-0.95), respectively. Across monthly intervals, the NER tool identified more potential clusters than were verified in WEDSS.

**Conclusions:**

We developed a novel pipeline of tools that identified existing outbreaks and novel clusters with associated addresses. Our pipeline ingests data from a statewide database and may be deployed to assist local health departments for targeted interventions.

## Introduction

As of December 1, 2021, the state of Wisconsin (WI) confirmed 884,701 cases of SARS-CoV-2 (COVID-19) [1]. At the county level, health departments use the free-text fields from COVID-19 initial case interview (contact-tracing) forms to identify potential businesses and facilities where transmission of the virus occurred and when individuals were infectious. During surges, public health workers encounter a high caseload and are overwhelmed with an abundance of free-text information in the interview forms. Current methods to mine the free-text fields are manual and a keyword-based approach, without rapid and systematic methods for finding cluster hotspots for targeted interventions (ie, guide risk communication, policy to limit capacity in certain businesses, compliance in enforcing orders at facilities and businesses). Methods in natural language processing (NLP) and machine learning have augmented workflows for COVID-19 care in other settings that are strained for resources and staffing [2-4], and may prove to be useful for health departments and their COVD-19 data teams that interact with the contact tracers and surveillance systems.

Named entity recognition (NER) is an NLP task to classify words according to a class, for example, identifying a token as a person, organization, or location. Current systems have leveraged the strength of pretrained neural language models [5] trained on a large corpus of data to achieve accuracy scores above 90% for NER tasks. Many of these systems are publicly available and have been fine-tuned to be run “out of the box” for applications, but there remains a paucity in the literature demonstrating its benefits in public health for outbreak surveillance work. Prior work in health care has demonstrated success in using pretrained neural language models for biomedical and clinical entity normalization [6] and building computable disease phenotypes [7]. The opportunities for public health providers and policy makers to leverage methods in NLP for data analytics is growing and becoming more accessible for nontechnical departments [8].

We aimed to develop an NLP pipeline that uses a pretrained NER neural language model applied to contact-tracing interview forms recorded in the Wisconsin Electronic Disease Surveillance System (WEDSS) to identify potential outbreaks during the COVID-19 pandemic. Further, we sought to design a novel location-mapping tool to identify the most likely address for a given named entity from our NER tool. The objective of our study was to measure the precision and recall for both the NER tool and the location-mapping tool in our NLP pipeline for identifying new clusters and existing outbreaks. Our pipeline may serve as a benchmark in public health informatics to assist contact-tracing efforts for targeted policies during COVID-19 and other pandemics, and provide scaled automation for state and local health department staff.

## Methods

### Data Source

WEDSS is a secure web-based system designed to facilitate reporting, investigation, and surveillance of communicable diseases, which includes data on COVID-19 since January 2020. WEDSS encompasses all of Wisconsin, but this study was a collaboration with Public Health Madison & Dane County (PHMDC), which serves the second-largest county in Wisconsin by population. Both structured and unstructured fields were extracted from WEDSS for our analyses, including the text fields from the county-level data containing relevant contact-tracing fields from the case interview forms. The case interview forms contained the followings sections: (1) symptoms; (2) laboratory and clinical information; (3) medical conditions; (4) COVID-19 risks, including travel; (5) residential and occupation settings; (6) potential sources of illness; (7) isolation and quarantine measures; (8) facility intervention; (9) contact-tracing details; and (10) investigation notes. The text fields included addresses for businesses, facilities, and schools where the exposed individual may have entered or worked. The investigation note field was the longest text field with a median token count of 127 (IQR 67-233) and frequently included dates and names of places visited by the individual during their exposure period. For the pipeline development, 26 structured and unstructured text fields from WEDSS data extracts were concatenated into 1 document as input into our language model. There was 1 document per case, and model runs were at the case level. Postprocessing of the named entities included the removal of frequently occurring named entities (ie, “Wisconsin,” “GMT”) identified from 12 months of posttesting case interview forms and removal of duplicate named entities.

Confirmed cases were individuals with a positive molecular or polymerase chain reaction (PCR) test result detecting SARS-CoV-2 RNA, filtered by the date of the test result. In alignment with the Centers for Disease Control and Prevention (CDC) case definition [9], individuals who tested positive were counted each time they had a new COVID-19 infection (defined as a positive test 90 days or more after their previous COVID-19 infection). Therefore, people may have been counted more than once, but this occurred in less than 1% of cases. Probable cases were cases not positive by a confirmatory laboratory test method (ie, PCR or molecular test) but met 1 of the following: (1) test positive using an antigen test method, (2) have symptoms of COVID-19 and a known exposure to COVID-19 (ie, being a close contact of someone who was diagnosed with COVID-19), or (3) have COVID-19 or SARS-CoV-2 listed on the death certificate.

There is no standard definition for a “cluster” or “outbreak” (the terms are interchangeable), and the CDC states the definition for outbreaks is relative to the local context [10]. Therefore, we followed the PHMDC definition for a cluster, which is 2 or more cases associated with the same location, group, or event around the same time [11], which we examined across 7-day intervals. Henceforth, we use the term “cluster” for a cluster identified from our NER tool and “outbreak” as the cluster that is identified and validated by the PHMDC COVID-19 data team following standard operating procedures and recorded in WEDSS.

### The NER Tool

We used a pretrained cased Bidirectional Encoder Representations from Transformers (BERT) base model [12], which was fine-tuned on the data set from the Conference on Computational Natural Language Learning (CoNLL)-2003 NER shared task [13]. This English data set remains 1 of the largest corpora in the public domain for NER, with 1393 Reuters news stories with a total of 35,089 annotated labels (5648 in the test set) across the categories of location, organization, person, and miscellaneous. The pretrained BERT model implemented in the Python Transformers library is maintained on the HuggingFace model repository [14-16]. The model reported an *F*_1_ score of 91.3, with a recall of 91.9 and a precision of 90.7, on the CoNLL-2003 test data set. At the time of this publication, the model represented the state of the art in NER [15]. We used the “out of the box” model and did not attempt to further fine-tune the models or adjust hyperparameters.

The text fields from WEDSS were preprocessed to remove nonmeaningful entities, such as contact tracers’ names. Postprocessing of the named entities included the removal of frequently occurring terms (ie, “Wisconsin,” “GMT”), the removal of duplicate named entities within 1 document, the removal of subword tokens that are occasionally tagged by the model, and the removal of patterns that were not informative. A WordPiece tokenizer was used to build groups of up to 512 tokens from each document, which were then fed into the model. For all case IDs from the extracted WEDSS data that had the same named entity reported, the average predicted probability was provided as the score for the likelihood of identifying it as a person, organization, location, or miscellaneous.

Entities found by the NER pipeline that were associated with an outbreak already discovered by contract tracers were identified through fuzzy matching. Known outbreak names and entities from the NER tool that shared an incident ID were also matched via the token sort ratio (each string to compare is tokenized and sorted alphabetically, and then similarity is calculated as similarity = [2 × number of matching characters/total number of characters] × 100). Entities and outbreaks with a token sort ratio of 70 or more were deemed to be matches.

### The Location-Mapping Tool

**Figure 1:**
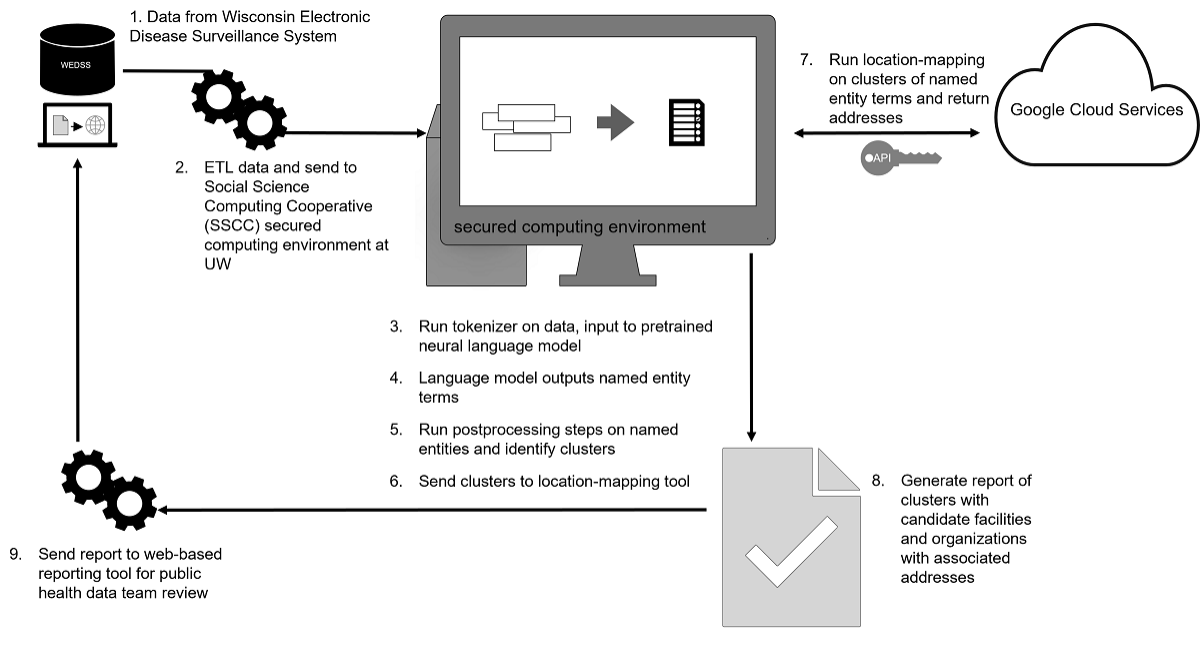
Process map for NER tool and location-mapping tool. ETL: extract, transform, and load; NER: named entity recognition.

**Figure 2:**
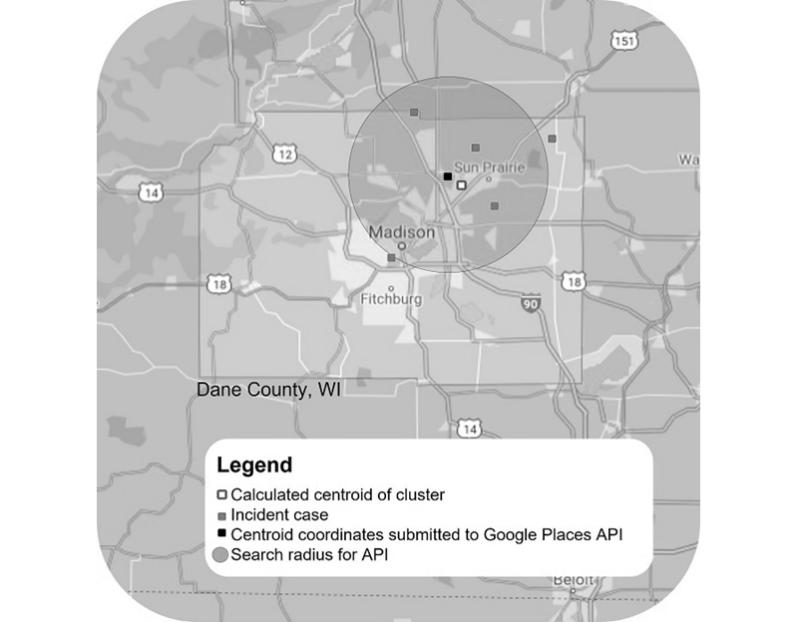
Location-mapping example for a cluster of COVID-19 clusters in Dane County as of October 2021. The grey dots show incident cases for a possible or known cluster/outbreak. The white dot shows the calculated centroid point of this cluster. The black dot shows the obfuscated centroid latitude/longitude point that is submitted to the Google Places API, which is shown by the larger gray circle. API: Application Program Interface.

During the development of our NER pipeline, we noted many named entities containing common business names that may have multiple locations within a county, such as “McDonald’s” or “Walmart.” Therefore, we developed a location-mapping tool into the pipeline using the Google Places Application Program Interface (API) to determine probable matches for locations that were near 1 or more case IDs within a cluster (Figure 1). The Google Places API requires searches to be within a circular zone with a maximum radius of 30 km. A sample search is shown in Figure 2. Multiple successive searches were permitted, although each search will increase API costs, and saturating a large search area with API calls would neither be optimal nor efficient. The commute distances for over two-thirds of the businesses in 36 major metropolitan areas in Wisconsin were between 0 and 24 miles [17], so 1 assumption of the mapping algorithm was that the named entity would be within commuting distance from the individual's home residence. Therefore, the individual’s latitude/longitude coordinates for each case ID in the cluster were extracted from WEDSS, and a k-means unsupervised approach was applied to identify the centroid coordinates from Google Places for the cluster of case IDs for a particular named entity.

The location mapper provided the most likely address for the named entity. If an exact match could not be made, then the top 3 results were filtered using fuzzy string matching. In either case, a predicted probability for each business address match was provided, and these results were then merged into a final report to provide an address associated with a named entity for use in contact tracing. Named entities that mapped to city names across the states of Wisconsin, Minnesota, Iowa, Michigan, and Illinois were filtered out because they were not specific enough to determine a precise location.

To comply with the Google Places API terms of service, we extracted the organizations and location names from the fields of all COVID-19-related forms in the WEDSS data to create an internal database of named entities for mapping between our NER tool and the Google Places API results. Therefore, no Google data were cached. The search algorithm only used the Google Places API to perform a search for named entities that matched our internal database of named entities. The full process map for the pipeline and reporting system is displayed in Figure 1.

The process map begins with the WEDSS data source and proceeds with an extract, transform, and load (ETL) procedure onto an on-premise Health Insurance Portability and Accountability Act (HIPAA)-secure computing environment at the University of Wisconsin (UW). The relevant fields from the case report interview forms go through feature engineering and the WordPiece tokenizer for the pretrained neural language model to classify named entities as business names and facilities. Only named entities that meet the criteria for a cluster with >2 incident IDs are sent to the location-mapping tool. The location-mapping tool identifies the centroid longitude/latitude of the cluster with a random shift for deidentification purposes. Next, the Google Places API is executed for the shifted centroid location, and proximity results of business and facility names are run against the named entities from the NER tool. An extended search radius is processed if no addresses are returned on the initial run. The top 3 results are shown from a fuzzy-matching schema with priority scores and shared in a report that is sent back to a web-based reporting system at the Wisconsin Department of Health Services (DHS). Any health department employee may view the report through the web-based reporting system.

### The Extended Location Algorithm

**Figure 3:**
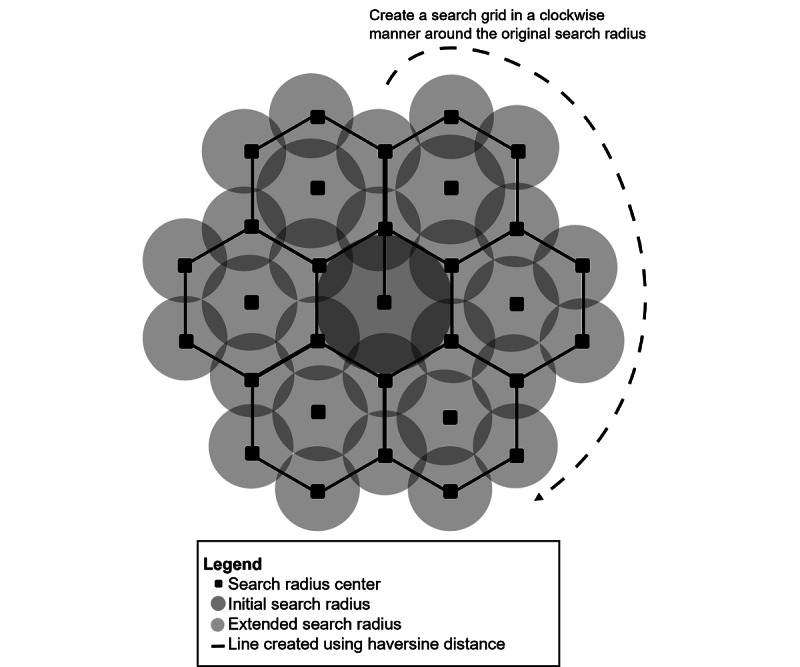
Framework for the extended location-mapping algorithm. If a named entity is not found within the initial search radius, additional search radii are created extending outward from the original search by creating a series of interlocking equilateral triangles, where each vertex of the triangle is a new API starting search point. The extended search stops when at least 1 match is found or the maximum distance is reached. API: Application Program Interface.

Some named entities that were submitted to the location-mapping tool were outside the 30 km search radius, but they may still be relevant for identifying novel clusters. For the named entities outside the search radius, we developed an extended search algorithm that covered a larger search radius and located business or organization names that would map to a given named entity not found within the initial 30 km search radius. The algorithm created and utilized a grid of interlocking equilateral triangles. The search grid first extended outward from the original latitude/longitude centroid point and then rotated in a clockwise manner around this centroid point, creating interlocking triangles (Figure 3). Each vertex of each triangle in the grid would become a new latitude/longitude starting point for an API call.

The curvature of the earth would mean that this grid would not follow straight lines, so we used an implementation of the haversine formula to create polygons with curved shapes in our grid [18]. Creating polygons by drawing outward would necessarily mean that the end of each rotation of the grid may not be the start of the rotation, so we set the search radii of each API call to overlap to ensure total coverage from the searches, and to account for this distance error between the starting point and ending point of the rotation. The extended searching stopped when either of the following occurred: (1) at least 1 search result was found by the API call, or (2) the grid reached a maximal range of 250 km from the original centroid latitude/longitude point.

### Evaluation of the NER Tool in the NLP Pipeline

All confirmed and probable individual cases in Dane County between July 1, 2020, and June 30, 2021, served as the retrospective validation data set. The NER tool was evaluated against confirmed outbreak facility/business names or valid business addresses linked to cases that were recorded in the WEDSS database. The named entities produced from the NER tool that met the criteria for a cluster (>2 instances of the named entity) within a 1-week period and matched a confirmed outbreak in WEDSS were labeled as true positives (TPs). In addition, named entities with >2 instances within a 1-week period that had a valid business address in WEDSS were also included in the TP group and represented novel clusters. The rationale to include both confirmed and unconfirmed outbreaks (potentially novel clusters) into the TP group was because they met the PHMDC definition for a cluster and contained a valid address that warranted an investigation (unverified outbreak) or avoided a redundant investigation (verified outbreak). Named entities that were produced from the NER tool without an address match in WEDSS were labeled as false positives (FPs). False negatives (FNs) were defined as confirmed outbreaks in the WEDSS data set that did not have a corresponding named entity from the NER tool. Evaluation metrics were report as precision = [TP/(TP + FP)] and recall = [TP/(TP + FN)]. These metrics are also known as positive predictive value and sensitivity, respectively. Evaluation of the NER tool was performed across a study period of 12 months. The precision and recall across the 12 months were reported to provide the largest sample size for reporting metrics. Monthly metrics were also reported to represent seasonal variation and various public health policies that affected case rates and prevalence, which would also affect precision [19].

### Evaluation of the Location-Mapping Tool in the NLP Pipeline

For the location-mapping algorithm, a separate set of precision and recall measures were reported. TPs were defined as a business address in our internal database of addresses from WEDDS that mapped to a Google Places API address for a named entity produced by the NER tool. An FP was a Google Places API address that did not map to the WEDSS database of addresses. An FN was defined as a named entity from the NER tool that mapped to our internal database of business names but returned no Google Places API address when a plausible API result could be found. Due to limitations on cost and computational resources, validation for the location-mapping algorithm was only performed for 1 month (October 2020). For both NER and location mapping validation, precision and recall measures were generated with bootstrapped 95% CIs.

### Report Generation for the COVID-19 Data Team at the Health Department

The goal of the pipeline was to generate a summary report from the contact-tracing forms collected in WEDSS across any time interval and identify potential clusters. A sample report is shown in Table 1 as a weekly report. Each cluster in the report also included the associated case IDs to guide the COVID-19 data team and the predicted probability for that cluster. Known outbreaks that were already identified by the COVID-19 data team or were under investigation were also extracted from WEDSS and included in the report to prevent redundancy in targeted policy efforts. The most likely address for each named entity was also provided from the location-mapping tool, along with a predicted probability.

**Table 1:** Example summary report for contact tracers for the county health department^a^.

Named entity^b^	Type	Frequency^c^	Predicted probability 1^d^	Case IDs^e^	Outbreak entity^f^	Address^g^	Predicted probability 2^h^
Sun Prairie	Place	12	0.67	12345, 12346	Sun Prairie	—^i^	100.0
Local retailer	Organization	7	0.54	12347, 12349, 22221	Retailer 001	Keys and Things, 21 Science Dr., Madison, WI	95.2
Big-box store	Organization	3	0.45	13347, 18349, 22221	Boxstore 08	Circuited City, 1561 Rocky Rd., Verona, WI	87.1
Fast-food place	Organization	2	0.71	17247, 18149, 29121	—	Burger Time 1234 State St., Madison, WI	88.2

^a^The example is based on fictitious data and not sourced from the original Wisconsin Electronic Disease Surveillance System (WEDDS) data due to privacy restrictions.

^b^Named entity: result from the named entity recognition (NER) pipeline. Named entities only qualified as cluster outbreaks if they had >2 case IDs associated with them.

^c^Frequency: unique mentions of NER across available case IDs from the reporting period.

^d^Predicted probability 1: average predicted probability from the classifier for the type of named entity.

^e^Case IDs: unique case IDs for lookup by the contact tracer.

^f^Outbreak entity: known outbreak exposures.

^g^Address: matched named entity using the longitude/latitude for the address from k-means clustering from Google Places Application Program Interface (API).

^h^Predicted probability 2: predicted probability from the location-mapping tool.

^i^No result from the NER or location-mapping tool.

The Institutional Review Board at the UW approved this study, and a data use agreement was established between the Wisconsin DHS and the UW. No data were shared outside the approved UW research environment and its approved users without explicit permissions by the UW and the DHS. The pipeline is currently available in the State of Wisconsin’s public health reporting system, and the source code is open source and publicly available [20].

## Results

### Characteristics of COVID-19 Cases and Noncases

**Figure 4:**
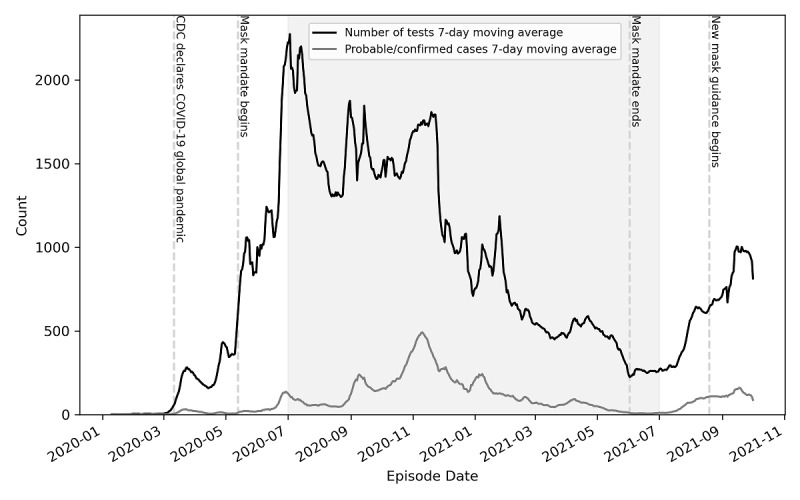
Trend over time of COVID-19 cases and noncases in Dane County, Wisconsin, between January 1, 2020, and October 31, 2021. Data are retrieved from the WEDSS. Line graphs of categories are aggregated by day and averaged in a moving 7-day window between January 2020 and October 2021. The CDC declared COVID 19 a global pandemic on March 11, 2020. The Public Health Madison and Dane County Initial mask mandate (Order 1) went into effect on May 13, 2020, and was updated and modified until June 2, 2021. Mandate 2 (Face-Covering Emergency Order) went into effect on August 19, 2021. The vertical dashed lines are demarcations for health policy changes. The gray-shaded area represents the validation period for our pipeline analysis. CDC: Centers for Disease Control and Prevention; WEDSS: Wisconsin Electronic Disease Surveillance System.

Of the 46,902 confirmed and probable cases, only 1595 (3.40%) were probable cases and the remainder were confirmed cases of COVID-19. In Dane County, non-Hispanic Whites accounted for 30,423 (64.87%) of the confirmed and probable cases, and the median age was 30 years (IQR 20-47); see Table 2. The most frequently reported occupation was student, but the missingness of the occupation variable in our WEDDS extract was high at over 75%. Additional demographics for the WEDSS data set are shown in Table 2. The 7-day moving average for cases and noncases in Dane County are shown in Figure 4 with delineation of the mask mandate policies between January 2020 and September 2021. The gray-shaded region represents the 12-month validation period in which we analyzed the NER tool for this study.

**Table 2:** Characteristics of COVID-19 cases and noncases in Dane County, Wisconsin, between July 1, 2020, and June 30, 2021.

Individual characteristics		Negative cases (N=323,424)	Probable/confirmed cases (N=46,902)	Total (N=370,326)
Age (years), median (IQR)		32 (20-51)	30 (20-47)	31 (20-51)
**Sex, n (%)**				
	Male	152,852 (47.26)	23,506 (50.12)	176,358 (47.62)
	Female	165,482 (51.17)	23,314 (49.71)	188,796 (50.98)
	Unknown	5090 (1.57)	82 (0.17)	5172 (1.40)
**Race/ethnicity, n (%)**				
	Non-Hispanic White	199,629 (61.72)	30,423 (64.87)	230,052 (62.12)
	Non-Hispanic Black	14,302 (4.42)	3266 (6.96)	17,568 (4.74)
	Hispanic	23,878 (7.38)	6662 (14.20)	30,540 (8.25)
	Other	85,615 (26.47)	6551 (13.97)	92,166 (24.89)
**Occupation, n (%)^a^**				
	Not recorded	311,809 (96.41)	37,083 (79.06)	348,892 (94.21)
	Nonuniversity student	3099 (0.96)	2391 (5.10)	5490 (1.48)
	University student	1161 (0.36)	903 (1.93)	2064 (0.56)
	Retired	573 (0.18)	468 (1.00)	1041 (0.28)
	Unemployed	502 (0.16)	429 (0.91)	931 (0.25)
	Other	6280 (1.94)	5628 (12.00)	11,908 (3.22)
**City, n (%)**				
	Madison	159,983 (49.47)	23,949 (51.06)	183,932 (49.67)
	Sun Prairie	22,667 (7.01)	3722 (7.94)	26,389 (7.13)
	Fitchburg	16,104 (4.98)	2983 (6.36)	19,087 (5.15)
	Middleton	15,991 (4.94)	1838 (3.92)	17,829 (4.81)
	Verona	15,224 (4.71)	1745 (3.72)	16,969 (4.58)
	Other	93,455 (28.90)	12,665 (27.00)	106,120 (28.66)

^a^Multiple responses were possible.

The validation data set comprised 4,183,273 total BERT tokens and 15,051 unique BERT tokens across the free-text fields in the contact interview forms. The longest field was “InvestigationNotes” with a median token count of 126.5 (IQR 67.0-232.5).

Across 12 months of validation, the recall was 0.67 (95% CI 0.66-0.68) and precision was 0.55 (95% CI 0.54-0.57). Of note, the precision and recall scores were variable from month to month as COVID-19 surges waxed and waned. The best performance was during surge months with a high volume of cases. Between October 2020 and January 2021, when caseloads ranged between 3300 and 7100, respectively, the recall was between 0.69 and 0.72, respectively. However, during months with fewer cases, such as between May 2021 and June 2021 (caseloads ranged between 149 and 410), the recall dropped down to 0.33 and 0.29, respectively. A similar trend was observed for precision, with a peak at 0.64 and a trough at 0.30 (Table 3). Across all months, the NER tool identified more potential outbreaks than were confirmed in WEDSS.

During the 1-month location-mapping tool validation period (October 2020), the *F*_1_ score was 0.93, with a recall of 0.93 (95% CI 0.92-0.95) and a precision of 0.93 (95% CI 0.92-0.95). There were 355 named entities that did not return a result for the Dane County search radius, but the extended location algorithm matched addresses in 202 (56.9%) of those named entities to our internal database of named entities for potentially novel clusters.

**Table 3:** Results from the NER^a^ tool by month for Dane County, Wisconsin, between July 1, 2020, and June 30, 2021.

Month	Cases, N	Confirmed outbreaks, n (%)	Total outbreaks identified by Automated Public Outbreak Localization through Lexical Operations (APOLLO), n (%)	Precision (95% CI)	Recall (95% CI)	*F*_1_ score
July 2020	508	137 (27.0)	251 (49.4)	0.51 (0.40-0.62)	0.28 (0.23-0.34)	0.37
August 2020	783	133 (17.0)	350 (44.7)	0.34 (0.25-0.44)	0.23 (0.17-0.29)	0.27
September 2020	2693	256 (9.5)	889 (33.0)	0.51 (0.46-0.56)	0.56 (0.51-0.59)	0.53
October 2020	4619	459 (9.9)	1267 (27.4)	0.64 (0.60-0.67)	0.69 (0.67-0.71)	0.66
November 2020	7129	564 (7.9)	1906 (26.7)	0.62 (0.59-0.65)	0.70 (0.68-0.72)	0.66
December 2020	3772	308 (8.2)	1078 (28.6)	0.58 (0.54-0.62)	0.70 (0.67-0.73)	0.64
January 2021	3361	241 (7.2)	1062 (31.6)	0.53 (0.49-0.58)	0.72 (0.69-0.75)	0.61
February 2021	2339	157 (6.7)	899 (38.4)	0.42 (0.36-0.47)	0.56 (0.51-0.61)	0.48
March 2021	1513	134 (8.9)	647 (42.8)	0.37 (0.31-0.43)	0.52 (0.46-0.57)	0.43
April 2021	1460	161 (11.0)	639 (43.8)	0.46 (0.39-0.53)	0.50 (0.45-0.55)	0.48
May 2021	410	81 (19.8)	233 (56.8)	0.41 (0.28-0.52)	0.33 (0.23-0.4)	0.36
June 2021	149	21 (14.1)	88 (59.1)	0.30 (0.12-0.52)	0.29 (0.11-0.44)	0.30

^a^NER: named entity recognition.

## Discussion

### Principal Findings

We developed a novel pipeline of tools that are able to extract large amounts of surveillance data and summarize a report to highlight existing and potential outbreaks and their associated addresses. The summary report was designed in weekly intervals and by county to identify outbreaks in a systematic approach for any region in the state of Wisconsin. We demonstrated the performance of our pipeline by focusing in Madison & Dane County, and we showed our pipeline performs best during high-case-volume periods when automated methods for contact-tracing efforts may be most needed. In addition, our pipeline has the potential to identify novel cluster outbreaks not identified by traditional methods. Ultimately, our tool may overcome existing limitations for data teams that need to build keywords and manually scan free-text reports for potential locations of outbreaks.

Tools leveraging methods in artificial intelligence have emerged for public health applications during the COVID-19 pandemic [21-23], and utilizing NLP for targeted policy efforts from contact-tracing data continues to be an area of interest as more tools are developed or become available [24,25]. Others have shown the benefit of pretrained neural language models for COVID-19 surveillance using nontraditional and unconventional public health data sources, such as Twitter feeds [26]. Informatics tools using more conventional methods have been developed using known contact details with a public health agency [25,27]; however, the study did not identify potential or novel outbreaks. We demonstrated the utility of an “out of the box” pretrained neural language model for NER to automatically scan the contact interview forms of COVID-19 cases and provide contact tracers with a simplified and organized summary report of existing and potential outbreak clusters. Our pipeline of tools follows the CDC guidelines for implementation and use of digital tools to augment traditional contact-tracing efforts [24]. As traditional approaches continue at the PHMDC and the state of Wisconsin to investigate and report positive cases into their surveillance system, our tool may help focus and guide data teams to clusters during high-volume caseloads. We also shared a novel location-mapping technology that uses the raw data from the state’s surveillance system and provides addresses to further reduce mining efforts from the larger databases.

The motivation for this work began with the COVID-19 data team at the PHMDC contacting the data science team at the UW to assist in methods to overcome the difficulties in mining the many free-text fields in the contact interview forms. Like other states and counties in the region, Dane County surged during the fall and winter months and our tool showed recall rates above 70% during these periods. Although the precision values were lower due to FPs, reviewing the FPs in our summary reports may still be less burdensome to data teams than manually scanning the free-text fields or building customized rule-based, keyword algorithms of individual reports. The more important determinant was reducing FNs of potentially missed outbreaks by having an acceptable recall. The accuracy of our tool dropped during months with caseloads below several hundreds, but we anticipate the tool may be less utilized during these periods because staff have more time to identify and investigate outbreaks through existing standard operating procedures. Currently, a version of the report has been incorporated into a statewide reporting system for beta testing and application across any county in the state of Wisconsin (Figure 1). First, the data from WEDSS are fed into the NLP pipeline via an ETL process in an on-premise, HIPAA-secure computing environment at the UW. The results are developed as a flat file output and returned to the state’s Department of Health Services Office of Health Informatics in a second ETL process into a statewide reporting system for access by end users at county health departments via a web interface. Our next steps are to examine acceptance by our PHMDC health department data team with pilot validation testing and to monitor for adoption, similar to what others have described [28]. We have integrated our pipeline to work in the existing statewide reporting system for counties.

Although the private sector with companies such as Google has led the field in geomapping technologies, we leveraged their location software to support our unique location-mapping tool. We remained compliant with license agreements by first building our own internal database of all potential business names and organizations derived from our WEDSS database. This allowed us to perform API calls and identify any matches without storing any of Google’s data and violating any license agreements to use the tool. We noted some of the NER places were chain restaurants and stores, so using unsupervised methods to identify a centroid longitude/latitude within a cluster allowed us to predict the most likely location of the business identified from the NER tool. Our precision and recall scores for this part of the system were high and potentially reduced the time needed by staff to identify exact addresses. Contact-tracing interview forms are collected at the county-level across the state of Wisconsin and recorded into the central WEDSS database, so we expect our tool may be scaled statewide to capture more rural regions or cover commute distances that span multiple counties.

### Limitations

Several limitations occurred in our work. First, we ran the NER tool as an “out of the box” solution without any further tuning. The training data set for fine-tuning a BERT-base-NER model came from a specific span of time and may not generalize well to our domain. However, due to time and resource constraints, we could not invest in building an internally annotated data set for fine-tuning. We expect model performance may continue to improve with domain adaptation, but we opted to develop our pipeline with the current general-purpose state-of-the-art tool. Second, we assumed the radius around the centroid of home addresses for clusters captured all relevant locations from our location-mapping tool. This does not account for individuals traveling from out of state or further distances from home. We did attempt to mitigate this issue with our extended mapping algorithm that had a radius of 250 km. Lastly, our tool was flexible for processing contact interview forms spanning different time intervals, but the delays in transfer of ETL data from the state of Wisconsin reporting system to PHMDC policy makers prevented a real-time alert system for the tool. Our current system can refresh every 24-48 hours from the time case report forms are entered into WEDSS, which remains useful to data analysts who are backlogged on reviewing cases during heavy-load periods. Our data use agreement prevented the real-time, on-site application of the pipeline by PHMDC staff, but this remains a future direction in data access and software development for our tool. Lastly, future work will incorporate results on the potentially new clusters to verify their relevance for investigation and confirm an outbreak. Prospective evaluation of the tool was not possible, given existing staff demands from the pandemic.

### Conclusion

Our automated pipeline ingests data from a statewide database, and it may be deployed across counties to assist other health departments in Wisconsin in targeted policies during outbreaks. The tool is open source and an interoperable resource that may be used by neighboring states as well. Further, our pipeline may also be applied for other communicable disease and surveillance efforts that requires analysis of free-text data.

## Abbreviations

API: Application Program Interface
BERT: Bidirectional Encoder Representations from Transformers
CDC: Centers for Disease Control and Prevention
CoNLL: Conference on Computational Natural Language Learning
DHS: Department of Health Services
ETL: extract, transform, and load
NER: named entity recognition
NLP: natural language processing
PHMDC: Public Health Madison & Dane County
FN: false negative
FP: false positive
TP: true positive
UW: University of Wisconsin
WEDSS: Wisconsin Electronic Disease Surveillance System
