# Correction: Identifying COVID-19 Outbreaks From Contact-Tracing Interview Forms for Public Health Departments: Development of a Natural Language Processing Pipeline

**DOI:** 10.2196/37893

**Published:** 2022-03-24

**Authors:** John Caskey, Iain L McConnell, Madeline Oguss, Dmitriy Dligach, Rachel Kulikoff, Brittany Grogan, Crystal Gibson, Elizabeth Wimmer, Traci E DeSalvo, Edwin E Nyakoe-Nyasani, Matthew M Churpek, Majid Afshar

**Affiliations:** 1 University of Wisconsin-Madison Madison, WI United States; 2 Loyola University Chicago Chicago, IL United States; 3 Public Health Madison & Dane County Madison, WI United States; 4 State of Wisconsin Department of Health Services Madison, WI United States

In “Identifying COVID-19 Outbreaks From Contact-Tracing Interview Forms for Public Health Departments: Development of a Natural Language Processing Pipeline” (JMIR Public Health Surveill 2022;8(3):e36119), the authors noted a few errors.

In the originally published paper, the following two sentences appeared under the “Results” section:

Of the 46,798 confirmed and probable cases, only 1588 (3.39%) were probable cases and the remainder were confirmed cases of COVID-19. In Dane County, non-Hispanic Whites accounted for 30,358 (64.87%) of the confirmed and probable cases, and the median age was 30 years (IQR 20-47).

These sentences have been corrected to:

Of the 46,902 confirmed and probable cases, only 1595 (3.40%) were probable cases and the remainder were confirmed cases of COVID-19. In Dane County, non-Hispanic Whites accounted for 30,423 (64.87%) of the confirmed and probable cases, and the median age was 30 years (IQR 20-47).

In the corrected version of the paper, Table 2 has been updated and can be viewed below. The originally published Table 2 is in Multimedia Appendix 1.

**Table 2:** Characteristics of COVID-19 cases and noncases in Dane County, Wisconsin, between July 1, 2020, and June 30, 2021.

Individual characteristics		Negative cases (N=323,424)	Probable/confirmed cases (N=46,902)	Total (N=370,326)
Age (years), median (IQR)		32 (20-51)	30 (20-47)	31 (20-51)
**Sex, n (%)**				
	Male	152,852 (47.26)	23,506 (50.12)	176,358 (47.62)
	Female	165,482 (51.17)	23,314 (49.71)	188,796 (50.98)
	Unknown	5090 (1.57)	82 (0.17)	5172 (1.40)
**Race/ethnicity, n (%)**				
	Non-Hispanic White	199,629 (61.72)	30,423 (64.87)	230,052 (62.12)
	Non-Hispanic Black	14,302 (4.42)	3266 (6.96)	17,568 (4.74)
	Hispanic	23,878 (7.38)	6662 (14.20)	30,540 (8.25)
	Other	85,615 (26.47)	6551 (13.97)	92,166 (24.89)
**Occupation, n (%)^a^**				
	Not recorded	311,809 (96.41)	37,083 (79.06)	348,892 (94.21)
	Nonuniversity student	3099 (0.96)	2391 (5.10)	5490 (1.48)
	University student	1161 (0.36)	903 (1.93)	2064 (0.56)
	Retired	573 (0.18)	468 (1.00)	1041 (0.28)
	Unemployed	502 (0.16)	429 (0.91)	931 (0.25)
	Other	6280 (1.94)	5628 (12.00)	11,908 (3.22)
**City, n (%)**				
	Madison	159,983 (49.47)	23,949 (51.06)	183,932 (49.67)
	Sun Prairie	22,667 (7.01)	3722 (7.94)	26,389 (7.13)
	Fitchburg	16,104 (4.98)	2983 (6.36)	19,087 (5.15)
	Middleton	15,991 (4.94)	1838 (3.92)	17,829 (4.81)
	Verona	15,224 (4.71)	1745 (3.72)	16,969 (4.58)
	Other	93,455 (28.90)	12,665 (27.00)	106,120 (28.66)

^a^Multiple responses were possible.

The correction will appear in the online version of the paper on the JMIR Publications website on March 24, 2022, together with the publication of this correction notice. Because this was made after submission to PubMed, PubMed Central, and other full-text repositories, the corrected article has also been resubmitted to those repositories.

## Appendix

Multimedia Appendix 1 Originally published Table 2.

