# Supplementary material for: Correction: Identifying COVID-19 Outbreaks From Contact-Tracing Interview Forms for Public Health Departments: Development of a Natural Language Processing Pipeline
Source: JMIR Public Health Surveill. 2022 Mar 24;8(3):e37893. doi: 10.2196/37893 (PMC8990338; doi:10.2196/37893)
Supplement: Multimedia Appendix 1 [file publichealth_v8i3e37893_app1.docx]

Table 2. Characteristics of COVID-19 cases and noncases in Dane County, Wisconsin, between July 1, 2020, and June 30, 2021.

| Individual characteristics | | Negative cases (N=321,383) | | Probable/confirmed cases (N=46,798) | | Total (N=368,181) | |
| --- | --- | --- | --- | --- | --- | --- | --- |
| Age (years), median (IQR) | | 32 (20-51) | | 30 (20-47) | | 31 (20-51) | |
| **Sex, n (%)** | | | | | | | |
|  | Male | | 151,947 (47.27) | | 23,449 (50.11) | | 175,356 (47.63) |
|  | Female | | 164,355 (51.15) | | 23,266 (49.72) | | 187,621 (50.97) |
|  | Unknown | | 5066 (1.58) | | 82 (0.18) | | 5148 (1.40) |
| **Race/ethnicity, n (%)** | | | | | | | |
|  | Non-Hispanic White | | 198,027 (61.63) | | 30,358 (64.87) | | 228,385 (62.04) |
|  | Non-Hispanic Black | | 14,238 (4.43) | | 3253 (6.95) | | 17,491 (4.75) |
|  | Hispanic | | 23,733 (7.39) | | 6644 (14.20) | | 30,377 (8.25) |
|  | Other | | 85,330 (26.56) | | 6542 (13.98) | | 91,872 (24.96) |
| **Occupation, n (%)^a^** | | | | | | | |
|  | Not recorded | | 309,824 (96.42) | | 37,045 (79.16) | | 346,869 (94.23) |
|  | Nonuniversity student | | 3081 (0.96) | | 2385 (5.10) | | 5466 (1.48) |
|  | University student | | 1157 (0.36) | | 902 (1.93) | | 2059 (0.56) |
|  | Retired | | 569 (0.18) | | 465 (0.99) | | 1034 (0.28) |
|  | Unemployed | | 491 (0.15) | | 422 (0.90) | | 913 (0.25) |
|  | Other | | 6206 (1.93) | | 5578 (11.92) | | 11,784 (3.20) |
| **City, n (%)** | | | | | | | |
|  | Madison | | 158,824 (49.43) | | 23,892 (51.05) | | 182,716 (49.63) |
|  | Sun Prairie | | 22,578 (7.03) | | 3717 (7.94) | | 26,295 (7.14) |
|  | Fitchburg | | 15,973 (4.97) | | 2973 (6.35) | | 18,946 (5.15) |
|  | Middleton | | 15,895 (4.95) | | 1832 (3.91) | | 17,727 (4.82) |
|  | Verona | | 15,124 (4.71) | | 1745 (3.73) | | 16,869 (4.58) |
|  | Other | | 92,934 (28.91) | | 12,638 (27.01) | | 105,572 (28.68) |

^a^Multiple responses were possible.
